# The Vectis

**Published:** 1869-08

**Authors:** 


### The Vectis.

Dr. Swayne, of the Bristol Hospital and Medical School, says of the *vectis*, that it acts both as a lever and a tractor, its chief advantage being the facility with which it can be introduced, in order to correct malpositions of the head. He has found it very serviceable in presentations of the brow, where, as is generally the case, it is found impossible to push up the forehead so as to allow the occiput to come down. By passing up the vectis over the chin, that part may be brought down, and the presentation altered into one of the face, which is in every respect more favorable.

Again, in face presentations, if the chin be turned towards the hollow of the sacrum and do not readily engage itself underneath the pubic arch, it may be brought around into that position with the vectis.

Also, when the head presents with the forehead in the anterior semi-circle of the pelvis, we may often succeed by means of the vectis in bringing that part round into the posterior semi-circle.

Where, however, it is necessary to assist delivery because the head is arrested during its descent through the pelvis, the vectis is a singularly inefficient instrument. For, if used as a tractor only, it is almost sure to slip if much traction be made; and, if applied, as a lever only, it is unsafe, unless we make the hand a fulcrum, and then very little power can be applied, because the hand is not a sufficiently fixed point.

Dr. Robert Barnes has pointed out the best method of employing the vectis, both as a lever and a tractor. He directs that it should first be applied over the occiput, and that part drawn down until it is lower than the vectis; next the vectis should be applied posteriorly over the forehead, and that brought down lower than the occiput; and on, until at last the head is brought down through the outlet of the pelvis. But the first result of these procedures is apt to be the conversion of the ordinary presentation of the vertex into one of the occiput, which is not nearly so favorable. The same thing is apt to happen when the forehead has been depressed by the vectis, so that it may well be questioned whether we do not impede nature more, in these cases, by rendering the presentation less favorable, than we assist her by our extractive efforts with the vectis. Swayne is convinced that when the head is arrested in its course, and instrumental interference has been decided upon, it is better to have recourse to the forceps at once. He now never uses the vectis, except to correct mal-presentations.—*British Medical Journal.*

